# Intrinsic Chemical Consequences of Interface Failure in Composite Insulators Under Electrical Stress: PD-Induced Degradation of Epoxy/Anhydride Matrix and the Role of Humidity

**DOI:** 10.3390/polym18131556

**Published:** 2026-06-23

**Authors:** Kexin Shi, Dandan Zhang, Zhiyu Wan, Lixue Chen, Zhaohua Lu

**Affiliations:** 1 School of Electrical and Electronic Engineering, Huazhong University of Science and Technology, Wuhan 430074, China; z5322494@zmail.unsw.edu.cn (K.S.); wanzhiyu@outlook.com (Z.W.); chenlixue@hust.edu.cn (L.C.); m202572579@hust.edu.cn (Z.L.); 2 Key Laboratory of Pulsed Power Technology, Huazhong University of Science and Technology, Ministry of Education, Wuhan 430074, China; 3 State Key Laboratory of Advanced Electromagnetic Technology, Huazhong University of Science and Technology, Wuhan 430074, China

**Keywords:** composite insulators, decay-like degradation, partial discharge, epoxy/anhydride thermosetting matrix, ambient humidity, oxalic acid, interface

## Abstract

This study investigates the decay-like degradation mechanisms of the matrix material in composite insulators, focusing on the pronounced influence of humid environments on partial discharge (PD) characteristics and degradation pathways. A sealed chamber discharge platform was established, integrating PD signal monitoring, surface characterization, and gas chromatography-mass spectrometry (GC-MS) with molecular network analysis to examine the synergistic effects of thermal influences from PD and active atmospheric particles at humidity levels of 0% RH, 50% RH, and 100% RH. Results show that dry conditions favor high-energy, low-repetition-rate discharges, promoting cleavage and recombination of high-bond-energy bonds (e.g., benzene rings and (α)C–O), yielding primarily long-chain carboxylic acids (C9 and above). In contrast, humid conditions shift to low-energy, high-repetition-rate discharges, with water vapor decomposition generating highly oxidizing hydroxyl radicals (·OH). These facilitate selective scission of lower-bond-energy (β)C–O bonds and deep oxidation, significantly increasing short-chain dicarboxylic acids—especially oxalic acid—whose acidity and water solubility are nearly an order of magnitude higher than in dry environments, becoming the dominant acidic products. The work demonstrates that many PD-generated organic acids act as intrinsic corrosive agents in insulating systems, independent of ambient nitric acid. This elucidates, at the reaction pathway level, how high humidity modulates PD to enhance corrosive acid production, providing a microchemical basis for understanding regional decay-like failure patterns in composite insulators.

## 1. Introduction

Composite insulators, leveraging their superior mechanical and electrical properties, play a pivotal role in modern high-voltage transmission systems. Their structure features a glass fiber-reinforced epoxy resin core rod as the primary load-bearing component, externally encased in a silicone rubber sheath for protective functions. With extended service life, a decay-like fracture failure mode characterized by significant degradation of the core rod’s epoxy resin matrix has become increasingly prominent, posing a severe threat to the safe operation of power grids [1]. Extensive dissection analyses of insulators exhibiting decay-like fracture indicate that internal air gap defects (such as at the core rod-sheath interface) induce partial discharge (PD) behavior under high electric fields, serving as the primary driver of the decay-like degradation process [2,3,4].

Partial discharge (PD) is a localized dielectric breakdown process accompanied by multiple coupled physicochemical effects. During discharge, energetic electrons generated within micro-discharge channels deposit energy into highly confined regions, producing transient thermal shocks. Meanwhile, ionization and dissociation of air molecules generate abundant reactive species, including radicals, ions, ozone, and excited molecules. Under the combined action of localized thermal effects and plasma-generated reactive species, irreversible chemical degradation can readily occur on the surface and near-surface regions of insulating materials. As an organic material, the epoxy resin matrix of composite insulator core rods contains numerous C–C and C–O bonds with relatively low bond dissociation energies, making it particularly susceptible to discharge-induced degradation and therefore a critical weak link within the composite material system.

From the thermal perspective, extensive research has investigated the degradation behavior of epoxy resins under elevated-temperature conditions. For epoxy/anhydride thermoset systems commonly used in composite insulator core rods, Neiman [5] and Campbell [6] analyzed pyrolysis products generated below 400 °C, focusing primarily on light gaseous products and anhydride-derived compounds. More recently, Wan et al. [7] employed pyrolysis–gas chromatography–mass spectrometry (PY–GC–MS) to systematically investigate thermal degradation over a broader temperature range (200–1000 °C). Their results demonstrated that thermal degradation is governed predominantly by thermally activated bond scission processes, beginning with anhydride crosslink breakage and progressing toward main-chain decomposition at higher temperatures, ultimately generating anhydrides, carboxylic acids, phenols, and various hydrocarbon products. These studies established the fundamental thermal degradation pathways of epoxy resin materials and provided important insights into degradation reactions controlled by bond dissociation energies and pyrolysis kinetics.

Although PD is not equivalent to conventional thermal degradation, localized high-temperature regions can develop within PD micro-discharge channels. According to gas discharge theory, the energy density within these channels can be extremely high, leading to transient local temperatures reaching several thousand Kelvin [8]. Furthermore, dielectric barrier discharge studies have shown that PD typically manifests as discrete filamentary channels with pronounced spatial non-uniformity, resulting in localized high-energy regions often referred to as discharge hotspots [9]. From a mechanistic standpoint, these transient hotspots can activate bond scission reactions similar to those occurring during high-temperature pyrolysis, indicating that thermal degradation studies provide valuable insight into the thermally driven component of PD-induced material degradation.

From the plasma-chemical perspective, PD environments involve not only localized heating but also the continuous generation of highly reactive species capable of initiating oxidation reactions. Schifani [10], Hepburn [11,12,13,14], Jestin [15], and Yoshiyasu [16] conducted dielectric barrier discharge experiments on anhydride-cured epoxy resins and demonstrated that PD exposure can induce oxidation of the epoxy matrix. Hudon et al. [17] further investigated bisphenol A-type epoxy resins under discharge conditions in a sealed environment, reporting the formation of surface droplets and crystalline deposits and identifying acidic degradation products such as nitric acid, glycolic acid, and formic acid. However, existing studies have mainly focused on discharge characteristics, electrical treeing behavior, macroscopic property changes, or qualitative observations of degradation products [18,19]. Even recent studies addressing PD-generated organic acids and their corrosive effects on glass fibers within composite insulator matrices [20] have rarely provided systematic and quantitative insights into degradation pathways and product evolution at the molecular level.

While considerable progress has been achieved in understanding thermal degradation and PD-induced plasma degradation, increasing field evidence suggests that environmental humidity may represent another critical factor governing the development of decay-like fracture failures. According to field statistics reported in [21], all 22 documented that decay-like fracture incidents occurred in humid or semi-humid regions, including 16 cases in humid regions and 6 cases in semi-humid regions, whereas no comparable failures were reported in arid or semi-arid areas. This pronounced geographical distribution strongly indicates a close relationship between elevated environmental humidity and the occurrence of decay-like degradation.

Humidity can participate directly in discharge-induced physicochemical processes by influencing discharge characteristics, reactive species generation, oxidation reactions, and hydrolysis pathways. Under realistic service conditions, water molecules may further couple with PD-generated reactive species, promoting synergistic oxidation and hydrolysis reactions that accelerate material deterioration and alter interfacial properties. Nevertheless, despite the apparent engineering significance of humidity, how humid environments regulate degradation pathways and degradation-product distributions at the molecular level remains poorly understood. This knowledge gap limits a comprehensive understanding of the mechanisms responsible for the high susceptibility of composite insulators to decay-like fracture failures in humid regions.

This study replicates the degradation processes of matrix materials under different humidity settings by using a sealed chamber PD experimental platform. Utilizing gas chromatography-mass spectrometry (GC-MS) in conjunction with molecular network analysis techniques, thorough qualitative and semi-quantitative assessments of the intricate degradation products produced after discharge are performed. This study elucidates the regulatory patterns of environmental humidity on the selection of matrix degradation reaction pathways by comparing PD characteristics, material surface alterations, and the types and yield distributions of degradation products under varying humidity conditions, with the objective of providing direct microchemical evidence for comprehending susceptibility to decay-like fracture failures in composite insulators in high-humidity regions.

## 2. Materials and Methods

### 2.1. Epoxy Synthesis

This study used epoxy resin predominantly composed of bisphenol A diglycidyl ether (DGEBA), methyltetrahydrophthalic anhydride (MeTHPA), and 2-ethyl-4-methylimidazole. DGEBA has an equivalent weight between 183 and 190 g/mol, whereas MeTHPA serves as the curing agent, containing an anhydride group concentration of at least 40.5%. The proportion of the curing agent to epoxy molecules is roughly 85%. A minimal amount of 2-ethyl-4-methylimidazole acts as the accelerator, with a dosage ranging from 2 to 7 parts per hundred resin (phr) for every 100 parts of epoxy resin.

**Figure 1:**
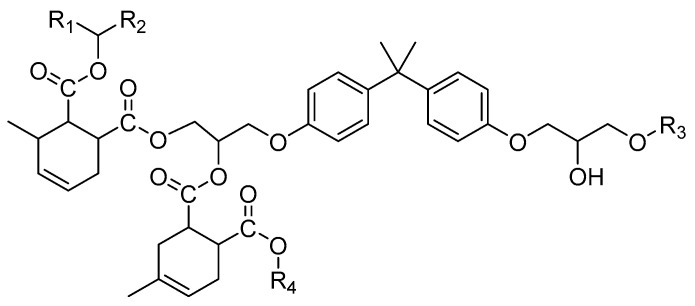
Schematic diagram of cross-linked epoxy thermoset matrix structure. Note: R1, R2, R3, and R4 denote generic alkyl or aryl substituents that are not central to the discussed PD-induced degradation mechanisms and humidity-related reactions.

After mixing the designated materials, a four-hour curing process was conducted at 120 °C, followed by natural cooling to ambient temperature. This curing composition and protocol were designed to promote efficient anhydride–epoxy copolymerization, yielding a thermoset network with high crosslink density, as confirmed by detailed PY–GC–MS analysis [7]. The use of 2-ethyl-4-methylimidazole facilitates the reaction between DGEBA and MeTHPA to form the crosslinked structure illustrated in Figure 1.

The resulting densely crosslinked network is expected to enhance degradation resistance by reducing free volume and thereby limiting the diffusion of reactive species (radicals, ions) and acidic degradation products under PD conditions. For clarity, the large molecular structures in Figure 1 are partially simplified using R1–R4 to represent portions of the molecules that lie outside the primary focus of this study.

In addition to defining the network structure, Figure 1 also provides a basis for subsequent analysis of PD-induced degradation mechanisms. The epoxy thermoset contains several chemically distinct bonds, including ester C–O bonds at the anhydride crosslinking junctions, aliphatic ether C–O bonds, aromatic ether bonds (Ar–O), aliphatic C–C bonds, and aromatic-ring C–C bonds. These bonds exhibit different bond dissociation energies (BDEs) (see Table 1) and therefore different susceptibilities to cleavage under discharge conditions.

The values listed in Table 1 represent typical ranges reported in thermochemical databases and literature. The exact bond dissociation energy depends on the local chemical environment and neighboring substituents.

### 2.2. Discharge Experiment Method

#### 2.2.1. Sealed Chamber Discharge Test Platform and Parameter Design

**Figure 2:**
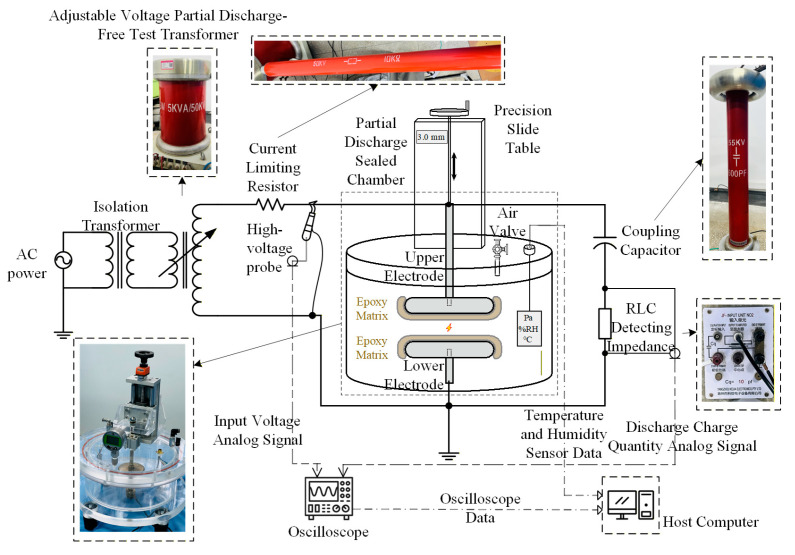
Schematic diagram of the designed sealed chamber discharge test platform for simulating internal air gap defect discharges in composite insulators.

To investigate the synergistic degradation effects of thermal influences and reactive species from PD on the core rod matrix material of composite insulators, a sealed chamber discharge experimental platform, as illustrated in Figure 2, was designed and constructed. This platform employs an isolation transformer and a PD-free test transformer to form the high-voltage power supply system, applying power-frequency alternating voltage between the upper and lower electrode plates in the chamber, with real-time monitoring of this voltage using a high-voltage probe. Within the sealed discharge chamber, two epoxy resin samples are positioned, and the air gap discharge between the upper and lower samples is utilized to simulate PD phenomena induced by internal air gap defects in materials under actual operating conditions. To ensure the comparability of discharge characteristics under different environmental conditions, all experiments were conducted under an identical applied voltage of 15 kV, while all other electrical and geometrical parameters of the discharge system were kept unchanged throughout the study.

The epoxy resin samples were prepared according to the process described in Section 2.1, with each sample having a thickness of 3 mm. A precision sliding stage was used to accurately adjust and stably maintain the distance between the upper and lower samples at 3 mm, achieving a control accuracy of ±0.02 mm. Consequently, the electrode configuration and discharge gap distance remained constant for all tests, ensuring that any observed variations in discharge behavior originated from environmental factors rather than changes in electric-field geometry. Although the macroscopic electric field in the experiment does not fully replicate the complex electric field distributions encountered in actual operations, the accelerated simulation setup within this sealed chamber effectively reproduces the thermal effects and atmospheric reactive species effects generated by PD, thereby providing an environmentally controllable experimental platform for studying the microscopic degradation mechanisms of the matrix material.

This study examines the impact of humidity—an essential experimental variable—on the evolution patterns of discharge behavior and the degradation reaction pathways of the matrix material, particularly in humid and semi-humid regions, while investigating the microscopic mechanisms of moisture’s influence. By including molecular sieve desiccants, saturated magnesium nitrate salt solutions, and deionized water within the chamber, three distinct atmospheric conditions were created: arid (0%RH), semi-humid (50%RH), and fully saturated humid (100%RH). Humidity was intentionally selected as the sole variable in this study; therefore, the applied voltage (15 kV), electrode spacing (3 mm), gas composition, specimen geometry, and test duration (20 h) were maintained identical for all experimental groups. These three representative levels were chosen based on: (1) field observations that decay-like fractures predominantly occur in humid and semi-humid regions with no reported cases in arid areas, indicating a strong humidity dependence [21]; (2) practical constraints of the sealed chamber platform, where precise long-term control at additional intermediate levels (e.g., 25% or 75%RH) would substantially increase experimental complexity and variability without proportionally enhancing mechanistic insights. PD studies were performed under each humidity level for a duration of 20 h. One of the primary aims of this study is to investigate trace degradation products; thus, the airtightness of the experimental platform is essential. The study employed the static approach of saturated salt solutions for humidity regulation.

To guarantee the airtightness of the experimental system, a standard gas cylinder was connected through an air valve before the experiment to fill the chamber with a standard mixed gas consisting of 78% nitrogen and 22% oxygen by volume fraction, while creating a slight negative pressure environment (pressure marginally below atmospheric pressure). The chamber remained static for one hour to assess its seal, during which a pressure sensor monitored pressure changes in real-time, guaranteeing that the amplitude of pressure fluctuations did not surpass 1%.

#### 2.2.2. Discharge Signal Monitoring Methods

The assessment of partial discharge signals was performed in compliance with the GB/T 7354-2018 standard [26], employing a detection branch consisting of a coupling capacitor and an RLC detecting impedance. The detector circuit was calibrated with a calibration pulse generator that produces standard pulses ranging from 50 to 500 pC.

The experiment utilized a Tektronix MDO3104 oscilloscope (Beaverton, OR, USA) (bandwidth: 1 GHz; maximum single-record length: 10 MS), which captured a full power-frequency cycle (20 ms) waveform every 30 s. The charge amount *q* for each individual discharge pulse and the total number of discharge pulses inside each power-frequency cycle were extracted from these waveforms.

The energy of an individual discharge pulse *W* was computed using Equation (1) [27]:

*W* = 0.5·*q*·*u*_i_ (1)

where *u*_i_ represents the applied voltage at the first discharge of the gap in each discharge cycle. The calculated energy values were normalized by the discharge area of a single specimen (approximately 78.5 cm^2^). Furthermore, the cycle-average discharge energy was defined as the total energy of all discharge pulses occurring within one power-frequency cycle divided by the corresponding number of discharge pulses, thereby characterizing the average energy level of discharge activity.

In addition, the discharge repetition rate (Hz) was calculated as the total number of discharge pulses detected within a recorded power-frequency cycle divided by the cycle duration (20 ms), representing the pulse occurrence frequency of the discharge activity.

#### 2.2.3. Analysis of Degraded Matrix Material and Products

(1) Characterization of Material Surface Functional Groups

Following PD exposure, the residues on the epoxy resin matrix were cleaned, and the material surface was subjected to functional group analysis using a Nicolet iS50R spectrometer (Waltham, MA, USA) via Fourier Transform Infrared Spectroscopy (FTIR). This study implemented a two-step processing approach to improve the accuracy and reliability of the spectral data. Initially, ATR correction based on Harrick theory [28] was conducted to reduce the impact of wavelength-dependent effective penetration depth in ATR measurements, ensuring that absorption peaks across all bands accurately represent the intrinsic absorption characteristics of the samples. Subsequently, polynomial baseline fitting was utilized to rectify the spectral baseline, removing background noise and baseline drift, thus enhancing the signal-to-noise ratio of the spectral data.

(2) Detection of Degradation Product Components

After PD exposure, gaseous epoxy degradation products present in the discharge space were collected in gas bags; liquid or crystalline products on the matrix surface were fully dissolved in methanol for detection. Both were analyzed for component separation and identification using GC-MS. The instrument employed was an Agilent 7890A/5975C (Santa Clara, CA, USA), with a DB-WAXetr highly polar polyethylene glycol (PEG) column selected as the chromatographic column. Compared to ion chromatography(IC), the gas chromatography–mass spectrometry (GC–MS) approach enables the separation and identification of a broader range of volatile and semi-volatile degradation products, including non-ionizable and structurally complex species, while providing detailed molecular-level structural information and relative product distributions. These capabilities are crucial for elucidating the detailed degradation pathways and assessing the chemical evolution of products under PD conditions.

To eliminate quantitative errors arising from variations in injection volumes, the mass spectrometric signal intensities were standardized and corrected. For gaseous products, the raw signal intensities were adjusted based on the ratio of the total gas volume to the actual injection volume; for liquid or crystalline products, they were first fully dissolved in methanol, followed by correction of the raw signals according to the ratio of the total sample volume to the injection volume.

The GC–MS data were first subjected to retention time (RT)-based deconvolution processing to eliminate peak overlap and background interference, thereby improving the resolution and identification accuracy of complex degradation products. The deconvoluted mass spectra were subsequently used to determine the chemical compositions and relative abundances of the degradation products.

Subsequently, a molecular network of degradation products was constructed using Cytoscape (version 3.10.3; Cytoscape Consortium, San Diego, CA, USA) based on the deconvoluted GC–MS data. The structural relationships among degradation products were evaluated according to mass spectral cosine similarity, and only associations satisfying the predefined similarity criterion were retained in the network. The resulting molecular network revealed structural correlations and potential transformation relationships among different products, thereby elucidating the degradation reaction pathways of the epoxy resin matrix under the synergistic effects of PD-induced thermal influences and reactive species.

To systematically evaluate the dominance of various degradation pathways under different experimental conditions, this study conducted a combined analysis of the yields and relative proportions of each degradation product component. Yields were quantified using the peak areas from the extracted ion chromatograms (EIC) of characteristic ions for each component, with changes directly reflecting the absolute generation efficiency of specific degradation pathways; relative proportions were defined as the percentage of a component’s peak area relative to the total peak area of all identified products, serving to reveal the relative importance and dominance of that component within the overall degradation product system.

Furthermore, molecular network relationships and quantitative product evolution characteristics were integrated to compare degradation pathways and evaluate their relative dominance under different experimental conditions.

It should be noted that, to prevent interference from the solvent methanol used in sample extraction on product identification and quantitative analysis, signals related to methanol were excluded and corrected during the data processing stage, ensuring that the obtained results accurately reflect the composition and characteristic changes in target products generated during the material’s thermal degradation process.

## 3. Results

### 3.1. Analysis of Discharge Process Signal Characteristics

**Figure 3:**
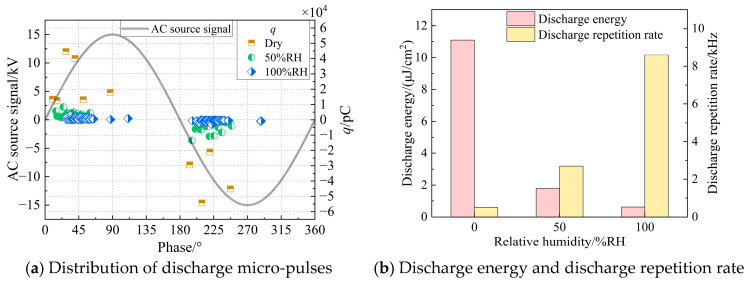
Effect of different humidity levels on discharge signal characteristics.

Figure 3 illustrates the impact of varying humidity levels on the properties of discharge signals.

The discharge event waveforms within a single power-frequency cycle under different humidity environments are shown in Figure 3a, where each scatter point represents a detected micro-discharge pulse. As evident from Figure 3a, the phase distribution of discharge charge quantities under varying humidities is predominantly concentrated in the 0–90° and 180–270° quadrants, consistent with the typical characteristics of partial discharge occurring in internal void defects of dielectric materials during the voltage rising phase [29].

Figure 3b depicts the discharge energy and discharge repetition rate across various humidity levels. Figure 3b illustrates notable variations in discharge modes and their macroscopic effects under different humidity levels. As humidity rises, the discharge energy demonstrates a distinct decline. As the relative humidity increases from a dry environment to 50%RH and 100%RH, the discharge energy diminishes to 16.22% and 5.59% of that seen in the dry environment, respectively. This behavior is chiefly ascribed to the energy consumption impact of moisture on electrons within the discharge channel in humid air. In arid environments, the diminished presence of water molecules in the air restricts energy dissipation for electrons within the discharge channel, facilitating a more efficient conversion of electric field energy into the kinetic energy of charged particles. This process engenders more robust electron avalanches and elevated ionization levels, ultimately resulting in greater micro-discharge pulse energies. In humid environments, the high polarity and electrophilicity of water molecules enable them to readily capture free electrons, significantly enhancing the effective attachment coefficient [30] and consequently diminishing air ionization efficiency, which reduces the availability of locally free electrons. Furthermore, charged particles like free electrons and ions are susceptible to adsorption or encapsulation by polar water molecules, resulting in the formation of bigger and more massive clusters of charged particles. This not only diminishes charge mobility but also prolongs their lifespan due to the protective shielding effect of water molecule clusters [31,32,33], significantly decreasing the likelihood of electron re-release during molecular collisions and thereby reducing new electron generation in the electron avalanche process. Concurrently, water molecules can mitigate photoionization effects through photon absorption [32]. These factors collectively result in heightened electron energy loss, diminished average energy, impaired ionization within the discharge channel, inhibited electron avalanche progression, and decreased discharge charge quantity, ultimately yielding substantially lower discharge energy relative to dry environments [34,35,36]. This process is also termed the quenching action of water molecules in pertinent literature [37,38].

On the other hand, Figure 3b demonstrates that while the energy of individual micro-discharges diminishes in humid conditions, the frequency of discharges significantly escalates with increasing humidity. When humidity rises from a dry environment to 50%RH and 100%RH, the discharge repetition rate escalates to 5.4 times and 17.2 times that of the dry environment, respectively. This occurrence is mostly attributed to two sources. Initially, due to the aforementioned quenching action of water molecules, the quantity of discharge charge in humid settings is reduced. Based on the fundamental mechanism of partial discharge under power-frequency voltage [39], the limited discharge quantity restricts the voltage drop across the air gap capacitance. Following the cessation of the discharge pulse, the residual voltage across the air gap capacitance stays elevated, necessitating a minimal alteration in the applied voltage to regenerate the discharge voltage, thus enabling expedited triggering of subsequent discharges and enhancing the repetition rate. Secondly, this phenomena may be associated with the markedly increased dissipation rate of local charges on the dielectric surface in humid conditions [34,35,36]. The charges accumulated on the insulating surface due to micro-discharge pulses are intricately linked to surface characteristics, especially surface conductivity. The adsorption of water molecules on the dielectric surface significantly increases surface conductivity, leading to the predominant attenuation of insulating surface charges via surface conduction [40], hence enhancing the migratory capacity of deposited charges. Deposited charges can diffuse or neutralize more swiftly through surface currents, expediting the charge dissipation process and facilitating charge distribution across larger insulating surface areas. Across an extensive area of the insulating surface, minor increments in applied voltage can elicit increasingly frequent low-amplitude discharge pulses. Conversely, in arid conditions, the surface conductivity of the matrix material diminishes, permitting charges to be localized, which facilitates the formation of bigger overvoltage and thus results in fewer but more energetic discharge pulses [34]. In conclusion, the electric field in humid settings can restore more rapidly to a threshold adequate for initiating subsequent discharges, resulting in a higher frequency of micro-discharge pulses and consequently a considerable increase in the discharge repetition rate.

In general, the discharge signal characteristics of arid and humid environments exhibit substantial differences. In arid conditions, the average discharge energy is elevated while the repetition rate is diminished; as humidity escalates, discharge energy progressively declines, however the repetition rate significantly increases. Studies by Wang [35] and Zhou [37] have documented analogous effects of humidity on discharge signal characteristics, indicating that as relative humidity incrementally rises from 8% to 29% and 77%, the partial discharge mode transitions from high-amplitude, low-frequency pulses to low-amplitude, high-frequency pulses. The previously reported experimental observations correspond with the theoretical explanation of discharge microscopic processes across varying humidity levels, thereby confirming the substantial influence of humidity on discharge behavior.

### 3.2. Changes in Gas Pressure During Discharge Process

**Figure 4:**
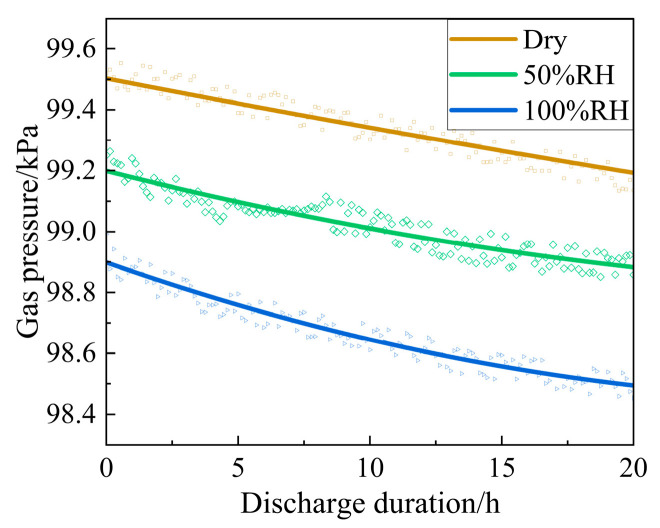
Variation curve of chamber pressure with discharge time at different humidity levels. Symbols represent the experimentally measured pressure data, while the solid lines denote the corresponding fitted curves.

Figure 4 depicts the temporal fluctuation of air pressure within the sealed chamber during the discharge experiment conducted at an ambient temperature of 15 °C. The symbols correspond to the experimentally measured pressure values, whereas the solid curves represent the fitted trends used to characterize the pressure evolution under different humidity conditions. The experimental data demonstrate that air pressure shows a consistent decline as discharge time increases. After 20 h of discharge experiments under varying humidity conditions, the air pressure diminishes by around 0.35 kPa. This experimental phenomenon corresponds with the observations documented by Holboll [41] and Schifani [42]. This pressure decrease indicates continuous consumption and transformation of gas-phase molecules during discharge, which is closely associated with the generation of reactive species such as ozone (O_3_), hydroxyl radicals (·OH), and oxygen radicals (O·). Although these gaseous species are not directly quantified due to detection limitations, their presence is widely recognized in air discharge environments and plays a critical role in initiating and sustaining oxidation and hydrolysis reactions on the material surface.

### 3.3. Degradation Characteristics of the Matrix Material Under the Synergistic Effects of Discharge-Induced Thermal Influences and Reactive Species

#### 3.3.1. Analysis of Surface Conditions of Epoxy Resin Matrix Material

**Figure 5:**
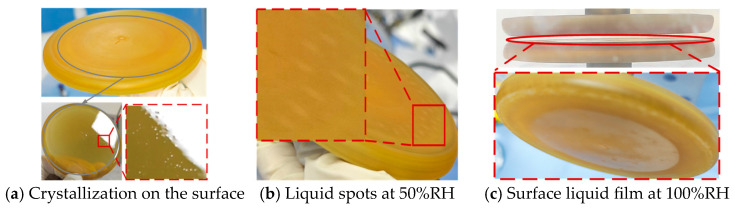
Comparison of the macroscopic surface morphology of epoxy resin matrix samples following 20 h of discharge under varying humidity conditions.

The surface states of samples under various humidity environments exhibited substantial differences following the conclusion of the discharge experiment (see Figure 5).

In the arid environment, the sample surface remained desiccated, with tiny crystals clearly and uniformly dispersed over the discharge zone, as illustrated in Figure 5a. In the 50%RH semi-humid environment, significant liquid patches emerged on the sample surface, as seen in Figure 5b. In the 100%RH humid environment, a consistent liquid layer developed in the central area of the sample, as represented in Figure 5c. This is mainly due to the increased adsorption of water molecules and the heightened oxidative effects of produced hydroxyl radicals in humid conditions.

#### 3.3.2. Changes in Chemical Functional Group Characteristics of Epoxy Matrix Material

**Figure 6:**
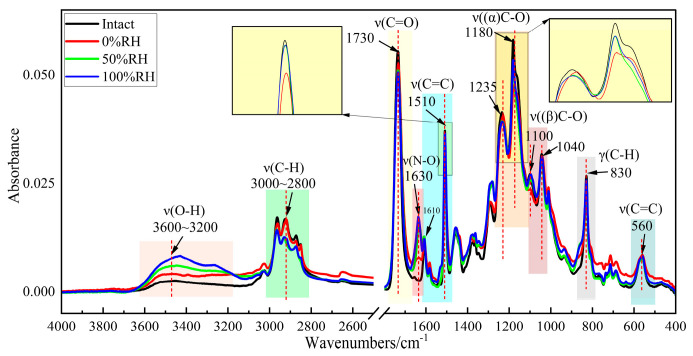
Infrared spectra of the epoxy resin matrix surface correlated with humidity at a consistent discharge duration.

Figure 6 depicts the alterations in chemical functional groups of the matrix material under consistent discharge periods with differing humidity levels. As summarized in Table 2 (with assignments supported by literature on DGEBA/anhydride-cured epoxy systems), the findings demonstrate that elevated humidity results in more significant reductions in the levels of methyl (–CH_3_) and methylene (–CH_2_) groups (3000–2800 cm^−1^), whilst the hydroxyl (–OH) content (3600–3200 cm^−1^) increases markedly, indicating a substantial enhancement in the degree of oxidation. This phenomenon principally arises from the potent oxidative effects caused by the significant production of hydroxyl radicals (·OH) in damp air. In humid environments, the concentrations of C=C bonds in the main-chain aromatic rings (1610, 1580, 1510 cm^−1^) and C–O bonds in aromatic ethers (1235 cm^−1^) demonstrate minimal variation compared to dry environments, indicating enhanced stability of the aromatic ring structures under humid conditions, potentially attributable to the elevated discharge energy in dry environments relative to humid ones. Moreover, at varying humidity levels, the concentrations of C=O carbonyl groups (1730 cm^−1^) and C–O bonds (1180 cm^−1^) in ester groups diminish, signifying the detachment and degradation of anhydrides. Notably, a distinct band emerges around 1630 cm^−1^ in humid conditions, tentatively assigned to N–O vibrations of nitrogen-containing species, indicating the involvement of nitrogen-containing oxidation products (e.g., NOx-derived species), which may contribute to nitric acid formation in humid environments. The synergistic effects of thermal influences from partial discharge and atmospheric reactive species damage the anhydride crosslinking points and main-chain structures in the crosslinked network of the core rod matrix material, while degradation results from the oxidative actions of oxygen radicals (O·) and hydroxyl radicals (·OH).

### 3.4. Analysis of Discharge Degradation Products

**Figure 7:**
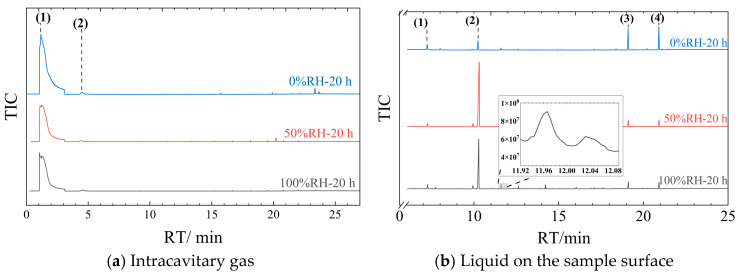
TIC profiles of collected products under different discharge humidity conditions.

Figure 7 illustrates the total ion chromatograms (TIC) of gaseous products produced by discharge (obtained from the discharge space) and liquid or crystalline products (extracted from the surface of the matrix material) under varying humidity conditions.

Figure 7a illustrates that the TIC of gaseous products displays two significant peaks at retention times (RT) of 1 min and 4.5 min, corresponding to peaks (1) and (2) in Figure 7a, respectively. The ion fragments corresponding to these peaks derive from atmospheric constituents, namely nitrogen (*m*/*z* 28), oxygen (*m*/*z* 32), carbon dioxide (*m*/*z* 44, 28, 16), and water vapor (*m*/*z* 18, 17). The concentrations of organic gaseous products from matrix degradation are minimal and fall below the instrument’s detection threshold, resulting in the lack of detectable mass spectral signals. Therefore, this study does not engage in additional examination of gaseous products in the discharge space.

Figure 7b demonstrates that the TIC of liquid or crystalline products produced by discharge on the matrix material surface exhibits prominent peaks during retention times of 7.24, 10.26, 19.11, and 20.92 min, corresponding to peaks (1) through (4), respectively. In humid conditions, the strength of peak (2) at RT 10.26 min significantly increases compared to the dry environment, while peaks (3) and (4) at RT 19.11 and 20.92 min noticeably diminish. Moreover, in humid conditions, the peak intensities at RT 11.6 and 12.63 min also increase substantially. This illustrates that, given equivalent partial discharge conditions, ambient humidity significantly affects the composition and yields of liquid or crystalline products.

### 3.5. Characterization and Mechanistic Analysis of Degradation Products Based on Molecular Network

Using the methodology described in Section 2.2.3, this study examined the influence of environmental humidity on the composition and yield of liquid or crystalline degradation products generated during the discharge process.

Three distinct molecular clusters were identified across all discharge experimental conditions: (1) Short-chain carboxylic acids with fewer than 4 carbon atoms (below C4) and alcohols; (2) Carboxylic acids with 4 to 6 carbon atoms (C4–C6); (3) Long-chain hydrocarbons with more than 9 carbon atoms and long-chain carboxylic acids (C9 and above). This signifies that the degradation products collectively demonstrate an acidic environment. To further examine the relationship between degradation-product evolution and discharge behavior, the GC–MS-derived product distributions were quantitatively compared with discharge characteristics, including discharge energy and repetition rate, under different humidity conditions. The results of this comparison are discussed in the following sections and provide additional evidence for the proposed degradation pathways and the role of discharge intensity in the degradation process.

#### 3.5.1. Short-Chain Carboxylic Acids

**Figure 8:**
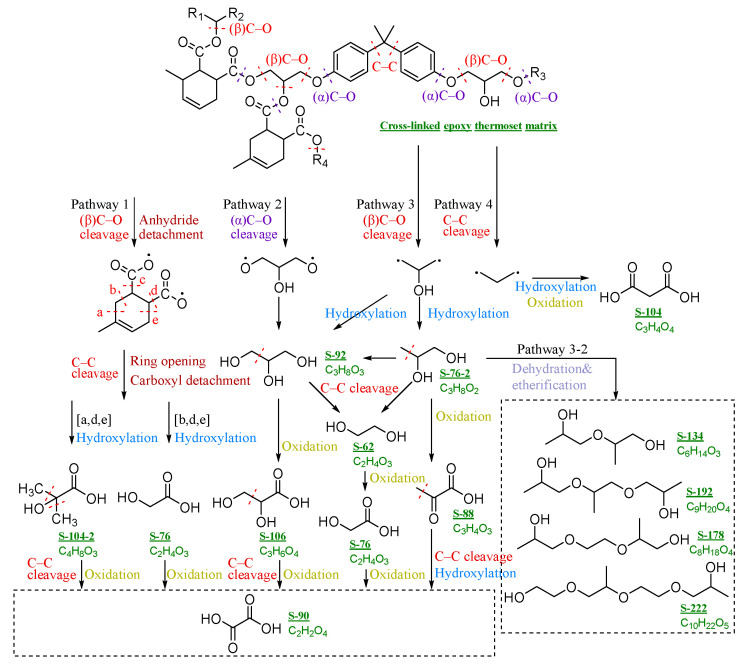
Reaction pathways for matrix degradation under the synergistic effects of discharge-induced thermal influences and reactive species, yielding short-chain carboxylic acids and alcohol products. Note: In ester groups (R–(C=O)–O–R’) or aromatic ether structures (Ar–O–R’), the (α)C–O bond refers to the carbonyl carbon–oxygen C–O bond or the aromatic ring–oxygen Ar–O bond; whereas the (β)C–O bond refers to the oxygen–alkyl O–R’ bond. Different colors are used solely to distinguish the reaction pathways and species. Arrows indicate the reaction direction. Lowercase letters and the dashed line represent cleavage of chemical bonds.

In the short-chain carboxylic acid fraction (RT approximately 9–12 min), malonic acid (oxalic acid, S-90) is the predominant compound. The alcohol fraction consists of alkyl alcohols (including S-90, S-62, S-76-2, RT ~ 7–8 min) and alkoxy alcohols (or ether alcohols, including S-192, S-134, S-222, S-178, RT ~ 15–18 min). An analysis of the product formation pathways and degradation reaction mechanisms of the epoxy resin matrix crosslinked polymer, considering its chemical structure and product types, is conducted under the synergistic effects of discharge-induced thermal influences and reactive species, with results illustrated in Figure 8.

It should be noted that the degradation pathways discussed here primarily focus on condensed-phase products identified by GC–MS. In actual discharge environments, gas-phase reactive species generated from air ionization (e.g., O_3_, ·OH, NOx) act as key initiators and oxidants, interacting with the polymer surface and driving the observed chemical transformations. The initial reaction pathway commences with the cleavage of the (β)C–O bond associated with the carbonyl at the anhydride crosslinking juncture in the epoxy resin matrix crosslinked polymer, resulting in the rupture of the crosslinking point and the formation of anhydride fragments. Additionally, the carboxyl group in the anhydride moieties dissociates, while the cyclohexene experiences C–C bond cleavage and ring opening. Hydroxylation occurs under the influence of hydroxyl radicals (·OH) produced by gas discharge, resulting in the formation of carboxylic acids S-104-2 and S-76. S-104-2 experiences C–C bond cleavage and demethylation, subsequently transforming into oxalic acid S-90 through oxidation by ozone or radicals in the discharge environment; S-76 is directly oxidized to oxalic acid S-90.

The secondary reaction pathway arises from the breakage of the (α)C–O bond in the carbonyl at the anhydride crosslinking juncture or inside the primary aromatic ether chain, resulting in the formation of glycerol S-92. S-92 undergoes additional oxidation to form carboxylic acid S-106 by the influence of ozone or radicals, subsequently leading to C–C bond breaking in S-106, followed by oxidation to produce oxalic acid S-90. Furthermore, S-92 can experience additional C–C bond cleavage under discharge energy, resulting in the formation of propylene glycol S-62; one hydroxyl terminus of S-62 is oxidized to yield carboxylic acid S-76, while further oxidation of the other hydroxyl terminus can additionally generate oxalic acid S-90.

The third chemical pathway results from the breakage and hydroxylation of the (β)C–O bond linked to the carbonyl at the anhydride crosslinking site or inside the main-chain aromatic ether, producing propylene glycol S-76-2. S-76-2 undergoes further oxidation to provide carboxylic acid S-88 and oxalic acid S-90. Furthermore, S-76-2 may participate in events akin to those in the second pathway, resulting in the formation of glycerol S-92, which is further reduced to carboxylic acid S-76 and oxalic acid S-90. Excessive concentrations of alkyl alcohols, such as S-76-2, in the system or low atmospheric moisture levels can lead to the partial dehydration of alkyl alcohols, resulting in the formation of ether alcohols (pathway 3-2), including S-134, S-192, S-178, and S-222.

The fourth reaction pathway commences with the detachment of isopropyl groups resulting from C–C bond cleavage in the main-chain aliphatic segments, thereafter undergoing additional oxidation by the synergistic action of hydroxyl radicals, oxygen radicals, or ozone to produce carboxylic acid S-104.

Pathways 1–4 predominantly yield diverse carboxylic acids and alcohols, with nearly all pathways ultimately capable of creating oxalic acid S-90 via oxidation processes or further bond cleavages. Consequently, among short-chain carboxylic acids, oxalic acid stands out as the product with the highest yield.

**Figure 9:**
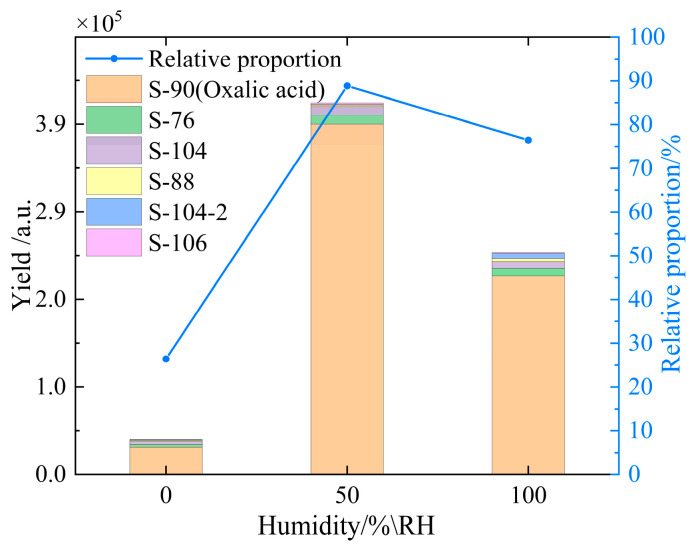
Changes in the yield (bar chart) and relative proportion (line chart) of short-chain carboxylic acids below C4 under different discharge humidities.

Figure 9 illustrates the peak area yields (in arbitrary units, a.u.) and the relative fraction variations in short-chain carboxylic acids containing fewer than four carbon atoms under varying discharge humidity conditions.

Figure 9 indicates that an increase in discharge ambient humidity markedly improves the yield of short-chain carboxylic acids containing fewer than four carbon atoms. This trend is consistent with the variations in discharge repetition rate and effective discharge energy under different humidity conditions, indicating a coupling relationship between discharge characteristics and chemical product formation. Under equivalent discharge periods, an increase in humidity from 0%RH to 50%RH results in a yield of short-chain carboxylic acids that is 9.66 times greater than that seen at 0%RH, with the relative proportion escalating from 26.43% to 88.85%. This is mainly due to the significant production of hydroxyl radicals from moisture ionization in humid conditions, which greatly improves the effectiveness of hydroxylation and subsequent oxidation in reaction pathways 1, 3, and 4, hence increasing the generation rate of carboxylic acids. Nevertheless, as humidity escalates from 50%RH to 100%RH, the yield of short-chain carboxylic acids diminishes marginally, a variation mostly associated with the influence of humidity on discharge properties. As outlined in Section 3.1, in identical electric fields, moisture in humid conditions diminishes the energy of individual discharge micro-pulses, resulting in reduced effective energy for cleaving C–C or C–O bonds during single discharge events, consequently decreasing the likelihood of successful bond cleavage. The examination of reaction pathways in this section reveals that the production of low-molecular-weight carboxylic acids necessitates several C–C bond cleavages; consequently, the diminished probability of bond cleavage during single discharge events leads to a decrease in the yield of short-chain carboxylic acids. However, the heightened frequency of discharge events in humid conditions somewhat counterbalances this by increasing the overall number of discharge occurrences per unit of time. Consequently, while the yield of short-chain carboxylic acids with fewer than four carbon atoms diminishes when humidity increases from 50%RH to 100%RH, the reduction is not significant. Under 100%RH circumstances, the absolute yield of short-chain carboxylic acids diminishes by a factor of 0.4 relative to 50%RH conditions, with the relative proportion declining by just 12.43%.

#### 3.5.2. C4–C6 Carboxylic Acids

**Figure 10:**
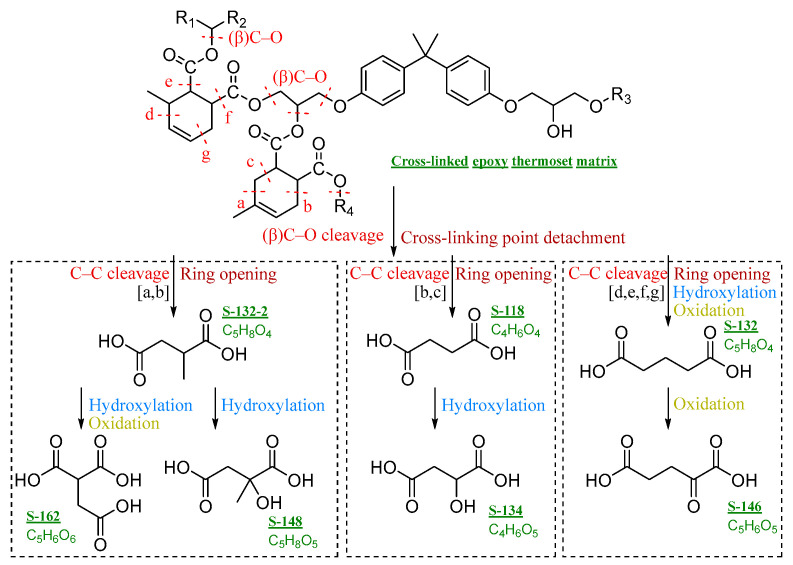
Reaction pathways for matrix degradation producing C4–C6 carboxylic acids under the synergistic effects of discharge-induced thermal influences and reactive species. Note: Different colors are used solely to distinguish the reaction pathways and species. Arrows indicate the reaction direction. Lowercase letters and the dashed line represent cleavage of chemical bonds.

Discharge breakdown products include clusters related to C4–C6 carboxylic acids, with RT of around 13–18 min. The analysis examines the product generation pathways and degradation reaction mechanisms of the matrix crosslinked polymer, considering its chemical structure alongside the structures and types of the products, under the synergistic effects of discharge-induced thermal influences and reactive species, as illustrated in Figure 10.

The chemical pathways for the synthesis of C4–C6 carboxylic acids via matrix discharge degradation predominantly stem from the breakage of the (β)C–O bond associated with the carbonyl at the anhydride crosslinking site, eventually resulting in the detachment of the anhydride crosslinking site. Then, C–C bond cleavage in cyclohexene facilitates ring opening, producing hydrocarbon radicals that then undergo hydroxylation and oxidation by oxygen radicals, resulting in the formation of diverse carboxylic acids.

As depicted in Figure 10, the cleavage of bonds a and b results in the ring opening of cyclohexene, directly producing carboxylic acid S-132-2, which may subsequently undergo hydroxylation or oxidation to form S-162 and S-148. Likewise, the ring opening caused by the cleavage of bonds b and c immediately yields carboxylic acid S-118, which subsequently undergoes hydroxylation to generate S-134. The cleavage of bonds d to g during ring opening subsequently produces carboxylic acid S-132 by hydroxylation and oxidation, which may further undergo oxidation to become S-146.

**Figure 11:**
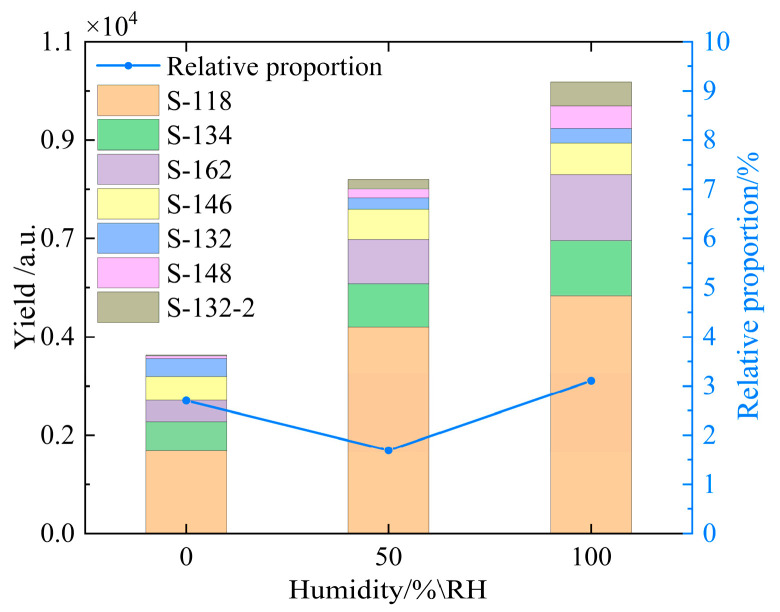
Changes in C4–C6 carboxylic acid yield (bar chart) and relative proportion (line chart) under different discharge humidity conditions.

Figure 11 illustrates the yields and relative proportion variations in C4 to C6 carboxylic acids under varying discharge humidity conditions.

Figure 11 indicates that an elevation in discharge ambient humidity markedly improves the yield of C4–C6 carboxylic acids. With equivalent discharge durations, an increase in humidity from 0%RH to 50%RH results in an almost doubling of the production of C4–C6 carboxylic acids. This occurrence corresponds with the trend noted for short-chain carboxylic acids containing fewer than four carbon atoms, principally due to the significant production of hydroxyl radicals from discharge in humid conditions, which hastens the synthesis of carboxylic acids. The relative fraction of C4–C6 carboxylic acids diminishes from 2.7% to 1.69%, primarily attributable to the significant rise in the yield of smaller molecules containing fewer than C4 atoms. As humidity escalates from 50%RH to 100%RH, the yield of C4–C6 carboxylic acids climbs to 1.53 times that seen at 0%RH, with the relative proportion advancing from 2.7% to 3.11%. In contrast to the minor reduction in yield for short-chain carboxylic acids with fewer than C4 atoms with increased humidity, the yield of C4 to C6 carboxylic acids persists in rising. The production of C4–C6 carboxylic acids necessitates fewer C–C bond cleavages, resulting in shorter reaction pathways and simpler formation. Consequently, despite diminished micro-discharge energy in high humidity, the heightened hydroxylation levels associated with increased humidity continue to facilitate the generation of C4–C6 carboxylic acids. These carboxylic acids may elicit secondary effects on glass fibers containing alkaline components within the core rod.

#### 3.5.3. Long-Chain Hydrocarbon and Carboxylic Acids

**Figure 12:**
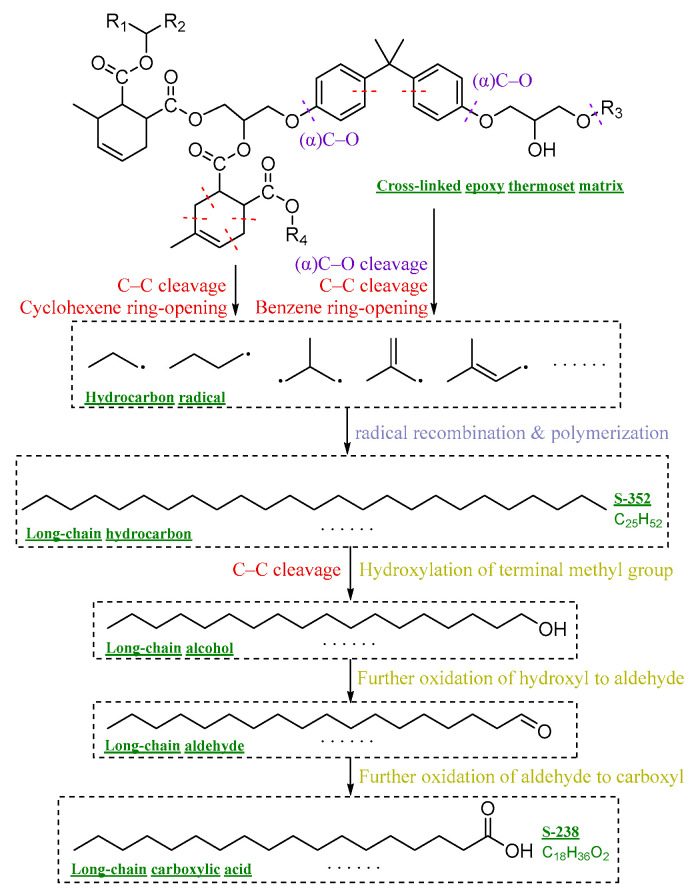
Reaction pathway for the breakdown of the matrix and the extension of long-chain carboxylic acids under the combined influence of discharge heat and active particles. Note: Different colors are used solely to distinguish the reaction pathways and species. Arrows indicate the reaction direction.

The molecular network derived from the discharge degradation products has two clusters linked to long-chain compounds. Among them, the long-chain hydrocarbon degradation products have RT of approximately 22–24 min, while the long-chain carboxylic acid degradation products have RT of approximately 11–20 min. The analysis of product generation pathways and reaction mechanisms of the matrix crosslinked polymer, considering its chemical structure alongside the structures and types of the products, is conducted under the synergistic effects of discharge-induced thermal influences and reactive species, as illustrated in Figure 12.

The cyclohexene or benzene rings experience ring-opening reactions due to high-temperature electrons produced by discharge, resulting in the cleavage of certain (α)C–O bonds, followed by subsequent recombination and polymerization to yield long-chain hydrocarbons of diverse molecular weights (e.g., S-252). Consequently, due to the action of oxygen radicals or hydroxyl radicals generated during the discharge process, the terminal methyl groups of long-chain hydrocarbons are replaced by hydroxyl groups, resulting in primary alcohols (R–CH_2_–OH). Excess oxygen in the experimental environment leads to the additional oxidation of primary alcohols by copious ozone or oxygen radicals in the discharge area, initially producing aldehydes, which are further oxidized to carboxylic acids (e.g., S-238).

**Figure 13:**
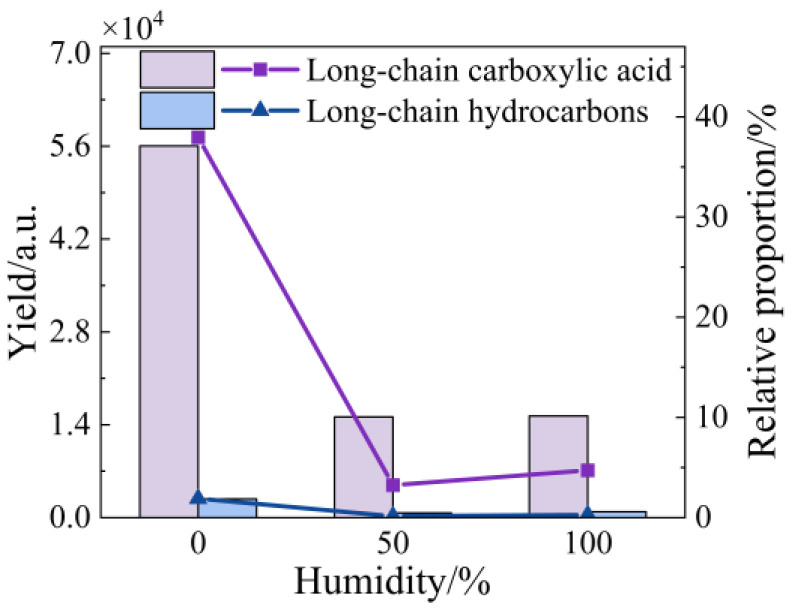
Yields of long-chain carboxylic acids and hydrocarbons at varying discharge humidity levels (bar chart) and relative proportions (line chart).

Figure 13 depicts the variations in yields (bar chart) and relative proportions (line chart) of long-chain hydrocarbons and long-chain carboxylic acids under various discharge circumstances.

Figure 13 indicates that an elevation in discharge ambient humidity markedly suppresses the production of long-chain hydrocarbons and long-chain carboxylic acids. Under identical discharge periods, when humidity reaches 100%RH, the absolute yields of these two product kinds diminish to 0.32 and 0.27 times their respective yields under dry conditions.

**Figure 14:**
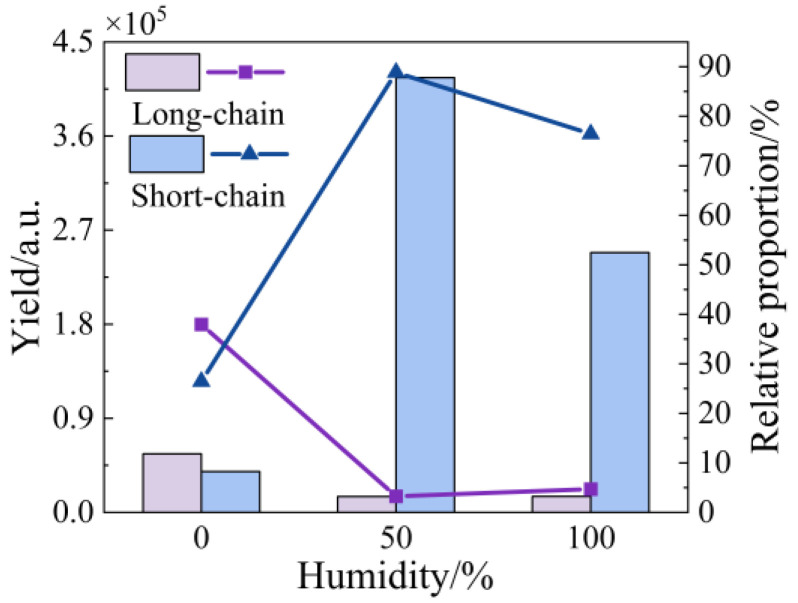
Comparison of yields of long-chain and short-chain carboxylic acids at various discharge humidity levels (bar chart) and their relative proportions (line chart).

Additionally, Figure 14 illustrates the yields (bar chart) and relative proportions (line chart) of short-chain carboxylic acids with fewer than four carbon atoms and long-chain carboxylic acids at varying humidity conditions for the identical discharge period. In arid conditions, the production of long-chain carboxylic acids is significantly higher, yielding 1.43 times that of short-chain carboxylic acids. Conversely, in humid conditions at 50%RH and 100%RH, the production of short-chain carboxylic acids, particularly oxalic acid, is markedly greater, yielding 27.39 times and 16.21 times that of long-chain carboxylic acids, respectively.

The observed phenomenon can be interpreted from the perspective of bond stability and humidity-induced changes in discharge characteristics. According to reported bond dissociation energies (BDEs) shown in Table 1, ester and ether C–O bonds located at anhydride crosslinking junctions generally exhibit lower bond dissociation energies (approximately 250–330 kJ mol^−1^) than aliphatic C–C bonds (approximately 340–370 kJ mol^−1^) [22,23,49], whereas aromatic-ring C–C bonds possess the highest stability (>500 kJ mol^−1^) [23,25] because of π-electron delocalization. As discussed in Section 3.1, increasing humidity reduces the energy of individual micro-discharge events. Consequently, cleavage of highly stable aromatic structures becomes less probable, while degradation preferentially occurs at ester and ether linkages. This interpretation is supported by the FTIR spectra shown in Figure 6, where the aromatic-ring-related signals remain relatively stronger under humid conditions, indicating improved preservation of aromatic structures. Moreover, the surplus of hydroxyl radicals in humid conditions renders small-molecule hydrocarbon radicals generated by discharge more prone to hydroxylation, obstructing their recombination or polymerization into long-chain compounds and consequently diminishing the yields of long-chain hydrocarbons and long-chain carboxylic acids; concurrently, the heightened discharge frequency in humid environments further facilitates the production of short-chain carboxylic acids, primarily oxalic acid.

#### 3.5.4. Discussion

Partial discharge leads to the degradation of the epoxy matrix, producing short-chain carboxylic acids with fewer than four carbon atoms (below C4), C4–C6 medium-chain carboxylic acids, and long-chain carboxylic acids and hydrocarbons with more than nine carbon atoms (above C9), thus creating an acidic environment that can induce secondary degradation of glass fibers. Conventional views ascribe acidic corrosion to externally produced nitric acid; however, this study establishes that organic acids resulting from matrix degradation constitute another significant endogenous acidic source, enhancing the comprehension of acid corrosion origins in the decay process.

Environmental humidity is a critical determinant affecting the choice of degradation routes and the distribution of products. Humidity fundamentally influences the selectivity of chemical bond cleavage by modifying the physical properties of discharge (energy and repetition rate) and the chemical milieu (types of reactive species). In arid conditions, the high-energy, low-repetition-rate discharge mode effectively surpasses the cleavage thresholds of high-bond-energy chemical compounds, including (α)C–O bonds in ester groups, aromatic ethers, and benzene rings. As a result, breakdown pathways are generally characterized by main-chain cleavage and ring-opening recombination, predominantly producing long-chain carboxylic acids and hydrocarbon compounds containing more than nine carbon atoms (see Figure 13).

Conversely, in humid conditions, discharge transitions to a low-energy, high-repetition-rate regime. Although the reduced energy of individual discharge events suppresses reactions involving high-BDE structures, the cumulative effect of repetitive discharges, together with the abundant generation of strongly oxidizing hydroxyl radicals (·OH), promotes preferential degradation of ester and ether linkages located at the anhydride crosslinking points. Subsequent hydroxylation and oxidation reactions redirect the degradation pathway toward crosslink rupture and oxidative fragmentation (see Figure 8), ultimately resulting in a nearly one-order-of-magnitude increase in the production of short-chain carboxylic acids represented by oxalic acid (S-90) (see Figure 9), which becomes the dominant acidic degradation product based on relative abundance.

It should be noted that the GC-MS results presented herein are based on relative peak areas (semi-quantitative analysis) and do not constitute absolute quantification of individual compound concentrations. Different degradation products (e.g., short-chain carboxylic acids versus alcohols or long-chain hydrocarbons) may exhibit varying detector response factors due to differences in ionization efficiency, fragmentation patterns, and matrix effects. Consequently, direct comparisons of absolute yields across chemically distinct species should be interpreted with caution. Nevertheless, within the short-chain carboxylic acid fraction (<C4), the observed relative trends-such as the marked increase in total peak areas with rising humidity from 0%RH to 50%RH (approximately 9.66-fold as reflected by peak area sums)-remain robust indicators of enhanced hydroxylation and oxidative pathways (Pathways 1, 3, and 4) driven by discharge-generated ·OH radicals. These semi-quantitative observations strongly support the proposed degradation mechanisms leading predominantly to oxalic acid (S-90) as the most abundant product based on relative peak area. For future kinetic modeling or cross-study comparisons, absolute quantification using internal standards (e.g., deuterated analogs) or calibration curves with authentic reference compounds would provide additional precision.

Consequently, moisture serves a dual function in decay-related degradation. At the chemical level, it selectively directs breakdown pathways towards the production of more corrosive short-chain carboxylic acids. On a physical level, it functions as a transport medium for corrosive substances. In arid conditions, the produced organic acids manifest as crystalline structures on the sample surface (refer to Figure 5a), rendering them immobile and hence hindering their ability to interact with and corrode the glass fibers within the core rod. In humid conditions, these acidic compounds dissolve in the water film adsorbed on the surface (see Figure 5b,c), resulting in a migratory acidic solution. This solution can infiltrate small flaws in the material, hence producing prolonged acid-etching effects on glass fibers within the core rod of composite insulators.

## 4. Conclusions

This study investigates the degradation behavior of the epoxy/anhydride matrix on composite insulator core rod surfaces under partial discharge (PD), with particular emphasis on the synergistic effects of discharge-induced temperature rise and reactive air species across dry, semi-humid, and humid conditions. Using a sealed-chamber PD platform, we systematically analyzed discharge signals, surface property changes, and degradation product evolution.

Key findings demonstrate that PD serves as the primary driver of matrix degradation by depleting atmospheric molecules and generating reactive species, with environmental humidity significantly modulating PD behavior and degradation pathways. In dry conditions, high-energy, low-repetition-rate discharges promote thermal-oxidative effects, leading to surface crystal formation and cleavage of aromatic and crosslinking structures. In humid conditions, increased discharge repetition rate and hydroxyl radical formation intensify oxidative and hydrolytic reactions, resulting in a continuous liquid film, elevated hydroxyl and nitro groups, and pronounced reduction in ester, methyl, and methylene contents. These humidity-dependent changes compromise the crosslinked network, which may lead to mechanical weakening and facilitate microcrack propagation.

Importantly, PD-induced degradation of the epoxy matrix generates endogenous organic acids (primarily short-chain carboxylic acids such as oxalic acid, C4–C6 acids, and long-chain acids >C9), whose types and yields are strongly influenced by humidity. These acids create a persistent acidic corrosive environment at the core rod-sheath interface, acting as a significant endogenous corrosion source that complements exogenous nitric acid mechanisms and accelerates decay-like deterioration. This independent corrosive action of organic acids is further substantiated by the previous detailed investigation [20], which revealed that organic acids are expected to play a significant and potentially dominant role under the investigated conditions, particularly due to their continuous in situ generation and localized accumulation through chelation-dominated mechanisms involving metal ion complexation and selective dissolution of the silicate network, while nitric acid acts as a complementary exogenous corrosive factor. This work provides new insights into the intrinsic chemical consequences of interface failure and underscores the need for humidity-aware material optimization in high-voltage insulation systems.

## 5. Challenges and Prospects

The sealed chamber discharge experimental platform developed in this study offers a well-controlled environment for examining the synergistic degradation effects of thermal influences and reactive species produced by partial discharge (PD) on the epoxy resin matrix of composite insulator core rods. Utilizing power-frequency AC voltage, precise air-gap adjustment (3 mm ± 0.02 mm), and controlled humidity levels (0%, 50%, and 100% RH), the platform effectively reproduces PD phenomena associated with internal air-gap defects and their secondary effects. The airtight design, achieved by filling with a standard gas mixture of 78% N_2_ and 22% O_2_ under slight negative pressure (pressure fluctuation <1%), ensures reliable capture and analysis of trace degradation products while minimizing external contamination. This setup is particularly valuable for investigating environmental influences prevalent in humid and semi-humid regions.

However, several challenges remain in translating these laboratory findings to real-world service conditions. The use of only three discrete humidity levels, while sufficient to reveal clear PD mode transitions and selective bond scission pathways, may not fully capture potential non-linear behaviors at finer gradients. Additionally, the simplified planar air gap and relatively uniform macroscopic electric field differ from the complex, non-uniform defects and multi-physics environments (shed geometry, surface contamination, dynamic humidity/temperature fluctuations) encountered in actual composite insulators after 5–10 years of operation. The current semi-quantitative GC-MS/molecular network analysis also faces limitations in detecting non-volatile or highly polar products, potentially underestimating secondary corrosive effects. In addition, the current analytical framework primarily focuses on condensed-phase degradation products, while gaseous products (e.g., O_3_, COx, NOx) are not directly detected due to instrumentation limitations. This may limit the completeness of the degradation pathway analysis, as gas-phase reactions and intermediates play a significant role in discharge-induced chemical processes.

Based on the observed chemical and structural evolution in this study, the degradation behavior also has important implications for the long-term mechanical and electrical performance of composite insulation systems. The cleavage of ester groups and anhydride crosslinking points, together with increased oxidation and hydroxyl content, indicates progressive deterioration of the crosslinked polymer network, which may weaken mechanical integrity and interfacial stability. Meanwhile, the generation and possible accumulation of acidic products, along with the introduction of polar functional groups (e.g., –OH and nitro-related species), may alter surface and interfacial electrical properties, potentially enhancing surface conductivity and facilitating partial discharge activity. These coupled effects are expected to accelerate insulation aging and contribute to failure phenomena such as interfacial degradation, microcrack propagation, and decay-like deterioration under prolonged service conditions.

While the platform demonstrates strong capability in isolating and accelerating key PD-induced mechanisms (thermal and chemical), opportunities exist to enhance the platform’s fidelity and broaden its applicability. Key areas for future research include:

- Refinement of defect morphology simulation: Current regular planar air gaps ensure repeatability for mechanistic studies. Future work could incorporate more realistic defects (irregular cavities, interfacial debonding, or microcracks) using 3D printing or field-aged samples to better replicate variations in PD inception voltage, pulse characteristics, and surface charge dynamics.
- Incorporation of multi-physics coupling: Advancing to finite element simulations that couple electric, thermal, and chemical processes would enable modeling of non-uniform field distributions influenced by shed geometry, core-sheath interfaces, and contamination. Integrating such simulations with experimental validation could better bridge laboratory results with real-world electric stress distributions.
- Expansion of environmental variables: While the current humidity gradients address a critical factor in wet regions, subsequent studies should extend the platform to coupled stresses such as temperature gradients, salt-fog/particulate pollution, UV exposure, and mechanical vibration. Developing multi-stress aging platforms would facilitate a deeper understanding of synergistic effects contributing to long-term phenomena like “lantern-shaped” core rod damage in humid service environments. In particular, finer humidity gradients (e.g., additional intermediate levels) could be explored in follow-up work to further validate trend reliability and non-linear behaviors.

## Figures and Tables

**Table 1:** Representative bond dissociation energies (BDEs) of chemical bonds relevant to the degradation of the anhydride-cured bisphenol-A epoxy thermoset.

Bond Type	Representative Structure	Typical BDE (kJ mol^−1^)	References
Ester C–O bond	R–C(=O)–O–R′	250–330	[22,23]
Aliphatic ether C–O bond	R–O–CH_2_–R′	280–330	[22,23]
Aromatic ether bond (Ar–O)	Ar–O–CH_2_–	330–380	[23,24]
Aliphatic C–C bond	–CH_2_–CH_2_–	340–370	[23]
Aromatic-ring C–C bond	Benzene ring	500–520	[23,25]

**Table 2:** FTIR Peak Assignments for Key Functional Groups in the Epoxy Matrix.

Wavenumber (cm^−1^)	Assignment	Literature Range (cm^−1^)	References
3600–3200	O–H stretching (hydroxyl)	3550–3200	[43,44]
3000–2800	C–H stretching (–CH_3_, –CH_2_)	2965–2850	[45,46]
1730	C=O stretching (ester/carbonyl)	1740–1720	[46,47]
~1630	N–O vibrations (nitro/nitrate)	1650–1600 (contextual)	[48]
1610, 1580, 1510	Aromatic C=C stretching	1615–1500	[43,45]
~1235	Aromatic ether C–O–C stretching	1250–1230	[43,46]
1180	Ester C–O stretching	1200–1150	[44,47]

## Data Availability

The original contributions presented in this study are included in the article. Further inquiries can be directed to the corresponding author.

## Abbreviations

The following abbreviations are used in this manuscript:

PD	Partial discharge
GC-MS	Gas chromatography-mass spectrometry
PY-GC-MS	Pyrolysis-gas chromatography-mass spectrometry
DGEBA	Bisphenol A diglycidyl ether
MeTHPA	Methyltetrahydrophthalic anhydride
RH	Relative humidity
FTIR	Fourier transform infrared spectroscopy
EIC	Extracted ion chromatograms
TIC	Total ion chromatograms
RT	Retention time
